# Identifying neuroimaging biomarkers for anxiety in emerging adults using machine learning and functional near-infrared spectroscopy during resting-state

**DOI:** 10.3389/fpsyt.2026.1722529

**Published:** 2026-04-10

**Authors:** Sibo Huang, Benguo Yu, Lijuan Liang

**Affiliations:** 1 School of Intelligent Medicine and Technology (Big Data Research Center), Hainan Medical University, Haikou, Hainan, China; 2 The First Clinical College, Hainan Medical University, Haikou, Hainan, China

**Keywords:** anxiety, biomarkers, functional near-infrared spectroscopy, machine learning, resting-state, SHAP

## Abstract

**Introduction:**

The neuroimaging biomarkers for anxiety diagnosis remain poorly understood. This study aims to identify cortical regions whose hemodynamic patterns, derived from resting-state functional near-infrared spectroscopy (fNIRS) combined with machine learning, may serve as potential biomarkers to assist in the assessment of anxiety cases.

**Methods:**

The final sample comprised ninety participants in emerging adulthood: 50 anxiety patients and 40 healthy controls (HC). After demographic data collection and Generalized Anxiety Disorder-7 (GAD-7) administration, all participants underwent a 1-minute baseline measurement and a 5-minute resting-state fNIRS recording. Following a stratified 70:30 train-test split, all feature selection procedures were performed using the training set. Model tuning and stability were assessed by five-fold stratified cross-validation within the training set, and final performance was evaluated on the independent test set.

**Results:**

The machine-learning GBDT model utilized 13 distinct features and exhibited enhanced efficacy in discriminating between patients with anxiety and healthy controls compared with the RF classification model typically employed for anxiety identification (AUC: 0.900 vs. 0.832, sensitivity = 0.921, and specificity = 0.709). SHAP-based interpretability analysis revealed that oxyhemoglobin (HbO) fluctuations in the prefrontal regions, particularly the dorsolateral/middle frontal/orbital middle frontal gyrus and sensorimotor-visual areas (precentral/middle occipital gyrus), emerged as potential predictors.

**Conclusion:**

The study provides a new perspective for the development of anxiety diagnosis tools and contributes candidate biomarkers for anxiety disorder identification and intervention.

## 1 Introduction

Anxiety, a prevalent mental disorder, is characterized by an exaggerated perception of potential threats, activation of defense mechanisms, persistent worry, avoidance behaviors, and autonomic dysregulation. Patients typically exhibit nervousness, impaired social functioning, reduced cognitive flexibility, and pronounced somatic symptoms, including sweating, frequent urination, dyspnea, and palpitations (1). According to a study that utilized data from the Global Burden of Disease (2), mental disorders are the leading cause of years lived with disability (YLDs), with YLDs increasing nearly five-fold from the 5–9 to the 20–24 age group (3). Globally, approximately 310 million individuals are affected by anxiety disorders (AD), with a heightened prevalence observed among individuals aged 15–29 (4).

The Medium- and Long-Term Youth Development Plan (2016–2025) explicitly emphasizes the importance of addressing youth mental health. Notably, the global prevalence of AD increased by 25.6% owing to the COVID-19 pandemic. Enduring adverse psychological effects significantly diminish patients’ quality of life and have bidirectional associations with various somatic diseases (5). An epidemiological study revealed that individuals with AD had a 1.7-fold higher risk of developing metabolic syndrome and a 58% higher incidence of cardiovascular disease (6). Moreover, patients with both anxiety and depression were more likely to engage in non-suicidal self-injury behaviors, with a 3.2-fold increased risk compared with those with a single disorder (7). Therefore, early and accurate diagnosis of AD, along with timely intervention, is essential for preventing symptom exacerbation.

The clinical diagnosis of anxiety relies predominantly on the Diagnostic and Statistical Manual of Mental Disorders, Fifth Edition (DSM-5) symptom criteria and subjective scale feedback. Owing to the subjective nature of patients’ self-reported symptom severity and the absence of objective biomarkers, the misdiagnosis rate is 42% during initial diagnosis (8). This challenge is particularly pronounced in primary care settings, especially in developing countries where the number of psychiatrists is fewer than 3 per 100,000 population, thereby hampering timely identification and tiered intervention. Consequently, developing an objective diagnostic system using biomarkers is crucial for precise intervention and treatment of anxiety patients (9).

Research revealed that aberrant cerebral cortex activity may indicate pathophysiological processes associated with mental illnesses. Both functional near-infrared spectroscopy (fNIRS) and functional magnetic resonance imaging (fMRI) revealed structural and functional disparities in the cerebral cortex of individuals with mental disorders (10). While fMRI is deeply rooted in neurobiological research, fNIRS is preferred in clinical settings due to its non-invasive nature. Compared to fMRI, fNIRS is more suitable for measuring the brain’s intrinsic spontaneous activation, owing to its high motion tolerance (up to 30 mm displacement error tolerance) and millisecond temporal resolution (sampling rate of 100 Hz) (11). A recent study demonstrated a delayed increase in the HbO signal in the dorsolateral prefrontal cortex (dlPFC) by 120–180 ms during mood regulation in patients with anxiety. This delay was negatively correlated with anxiety severity. However, fMRI may not reliably capture neural signals in such dynamic paradigms owing to a 5–8 second blood flow delay and associated risk of claustrophobia (15% incidence rate) (12).

fNIRS is user-friendly, noise-resistant, and safe to operate, which enables the monitoring of anxiety-related neural activity in ecologically valid settings like schools. This provides a distinct advantage for building objective diagnostic models (13). Consequently, its integration into clinical environments can aid in observing hemodynamic variations in patients with psychiatric disorders during cognitive tasks (14). However, previous studies have two limitations. First, most models were constructed using task-state data (e.g., verbal fluency tasks), which may introduce bias from task execution or individual cognitive discrepancies. Second, the lack of model interpretability impeded systematic analysis of the role of dynamic changes in HbO levels in crucial brain regions, which restricts the clinical translational potential of these potential biomarkers.

This study aimed to overcome the limitations of conventional neuroimaging techniques in analyzing groups that often overlook individual characteristics and fail to accurately predict responses to treatment. Therefore, a novel framework based on machine learning for personalized analysis was proposed. Resting-state fNIRS was used to derive hemodynamic features based on HbO. These features were used to identify clinically significant biomarkers, which circumvented the potential confounding effects of task-related neural activity patterns (15). Previous studies highlighted the potential of resting-state fNIRS with machine learning to detect psychiatric disorders. Studies demonstrated the feasibility of developing classification models for conditions, such as schizophrenia, by analyzing multichannel hemoglobin time-series data or different feature combinations to differentiate individuals with depression (16, 17). This study utilized the Shapley Additive Explanations (SHAP) interpretability framework to conduct feature-level attribution analysis for machine-learning model predictions, such as XGBoost (18). This study visualized the impact of HbO changes in anxiety-related brain regions, such as the frontal lobe, central sulcus, and occipital lobe, on the classification outcomes to enhance the model’s biological plausibility. We focused on feature engineering and the development of an optimal model classifier using resting-state fNIRS data with a limited sample size. Furthermore, we integrated SHAP summary plots and partial dependence plots and sought to elucidate the relationship between key neural biological features of brain regions and anxiety symptoms.

## 2 Materials and methods

### 2.1 Participants

This study recruited 133 university students aged 18–24 years enrolled at Hainan Medical University between July 2024 and January 2025. All participants had experienced anxiety symptoms for the first time and had not undergone any prior pharmacological treatment. Each participant completed the GAD-7 assessment (19), and those with GAD-7 scores ≥5 were recruited in accordance with international standards. Of these participants, 75 and 58 were categorized into the anxiety and healthy controls (HC) group, respectively. The experimental design demonstrated good reliability and validity.

Exclusion criteria comprised a history of: substance use (drug/tobacco/alcohol abuse); psychotic or other psychiatric disorders; epilepsy, intellectual disability, or organic brain disease; or a family history of major mental illness. Recent psychiatric care (within 24 weeks) or acute drowsiness/fatigue on test day also led to exclusion. All participants underwent psychological assessments and fNIRS measurements at the outpatient clinic of the First Affiliated Hospital of Hainan Medical University. They were native Chinese speakers with sufficient comprehension and communication skills and capable of effective cooperation. The final analysis comprised 50 patients with anxiety and 40 HC. This study was approved by the Ethics Committee of the First Affiliated Hospital of Hainan Medical University. Furthermore, written informed consent was obtained from each participant and their parents.

### 2.2 fNIRS recording

Stringent light-shielding measures were applied to the optodes. Simultaneously, participants were positioned in a noise-free setting to mitigate external disturbances (20). The experiment commenced with a 60-second pre-experimental baseline recording, during which participants were instructed to remain still and relax to establish stable cerebral oxygenation levels. Following this baseline period, the formal resting-state data acquisition comprised five consecutive 60-second blocks. During all blocks, participants were instructed to keep their eyes closed, remain still, and avoid systematic thinking.

**Figure 1:**
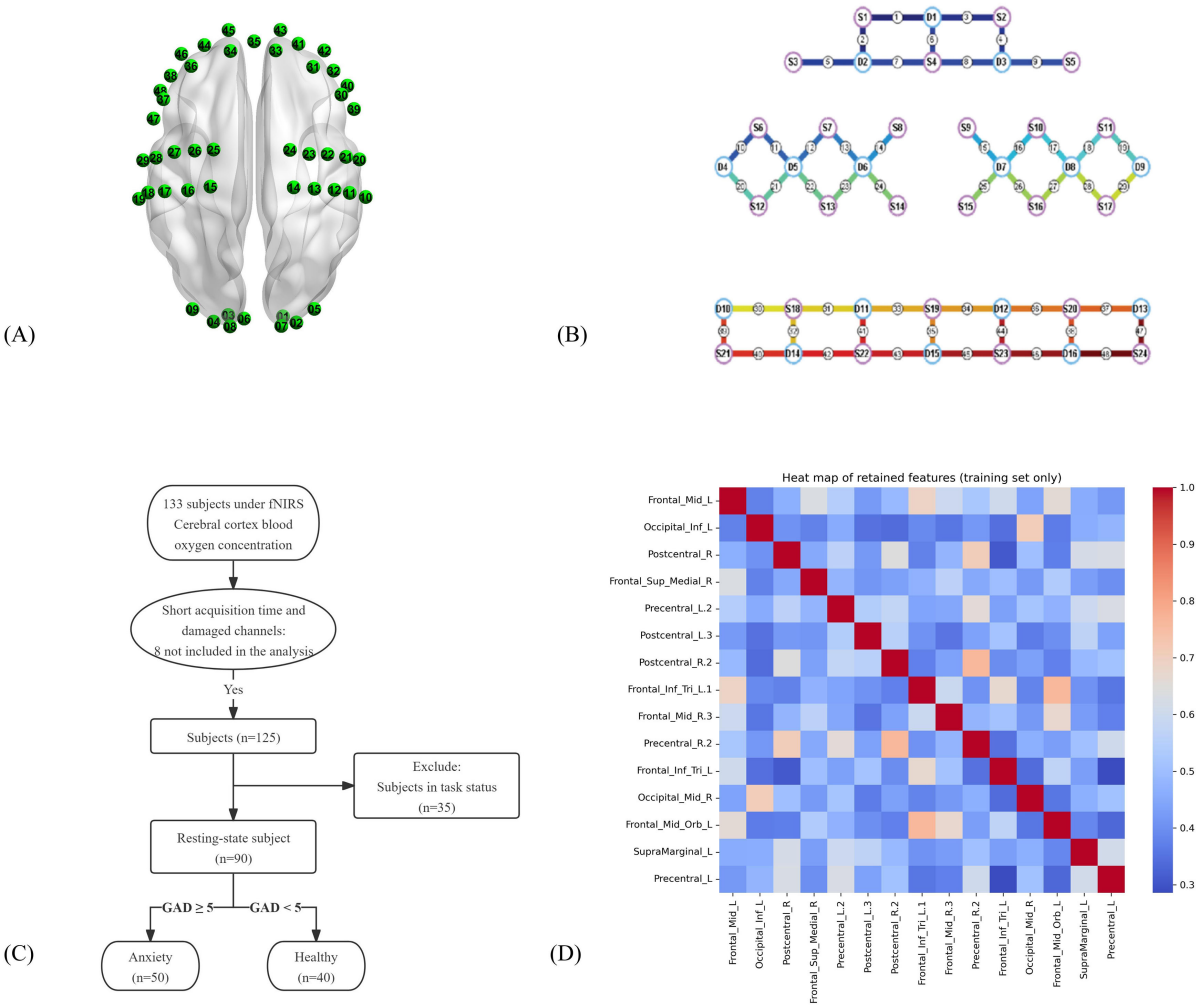
fNIRS measurements and multicollinearity mitigation. **(A)** fNIRS measurement channel map. **(B)** Detector probe distribution map. **(C)** Participants’ flow selection chart. **(D)** Spearman’s correlation analysis heat map.

Experiments utilized the NirSmart system (NirSmartII-3000A, Huichuang Medical Equipment Co., Ltd., Danyang, Jiangsu, China), employed in previous research (21). This system continuously monitored and recorded variations in HbO and deoxyhemoglobin (HbR) levels in participants at rest. It comprised near-infrared light sources, including light-emitting diodes (LEDs), and avalanche photodiode (APD) detectors. Light source probes emitted light at 730–850 nm wavelengths, with a sampling rate of 11 Hz. Acquired coordinates that followed the standard international 10/20 electrode placement system were converted into MINI coordinates and mapped onto the MINI standard brain template via the spatial registration method provided by NirSpace (Huichuang Medical Equipment Co., Ltd). Near-infrared light sources and detectors were positioned at 3 cm intervals. Links between adjacent light sources and detectors were termed “channels.” In this study, the system’s 48 channels comprised 24 light sources and 16 detector probes (**Figures 1A, B**), which encompassed brain regions in the frontal, parietal, occipital, and temporal lobes, based on the Brodmann areas.

### 2.3 Data preprocessing

During the fNIRS data extraction process, we excluded the time series and channels that exhibited abnormal signal corruption. Analysis was predominantly concentrated on HbO data owing to its more direct reflection of cognitive activation compared with deoxygenated and total hemoglobin. This proposition was substantiated by the higher correlation noted between HbO data and signals detected by the fMRI (22). This study employed Python (version 3.11.4), the pandas library (version 2.2.3), and the numpy library (version 1.26.4) to eliminate outliers, impute missing values, and preprocess raw data. Variables with > 25% missing data were excluded, and median imputation was used for the remaining missing values.

The dataset comprised 90 samples (**Figure 1C**). For each participant, the raw time series included a 60-second baseline and a subsequent 300-second resting period. Preprocessing involved truncating unstable segments at the onset and offset (typically 10–30 seconds in total) to ensure signal stability. Following this, the analyzable data length was standardized, yielding approximately 3,429 sampling points per channel for final feature extraction. A fixed-window segmentation method was utilized to process the NIRS signals with a window length of 60 s (23). This method preserved the original signal characteristics’ relative stability, which prevented the model from overemphasizing long-term sequences at the expense of local feature representation. Consequently, continuous NIRS signals were divided into 515 fragments that represented changes in HbO within specific time windows; these were structured into feature vectors as input to the classification model. Wavelet transformation within the 0.01–0.2 Hz range was applied to eliminate interference from higher-frequency noise, such as heart rate and respiration. The Modified Beer-Lambert law was employed to convert optical signals into hemodynamic parameters (24).

### 2.4 Feature selection

Feature selection was performed strictly within the training set, which was obtained by stratifying the entire dataset into 70% training and 30% test subsets prior to any feature engineering.

To mitigate collinearity that resulted from high data dimensionality, we initially conducted a Spearman’s correlation analysis on variables with a missing rate < 25% (25). If a variable has a correlation coefficient r > 0.8 with another significant variable, these variables should be evaluated based on their p-values. Additionally, if the variance inflation factor (VIF) exceeds 5, the variable is considered redundant and should be removed, thereby allowing the model to focus on predictors with greater discriminatory power. Chi-square scores were used as a ranking criterion, and the top 30 features were retained for downstream analysis. The chi-square p-values are provided for transparency but were not used as a strict inferential cutoff.

We also utilized mutual information analysis to determine the feature and target variables’ mutual information values and evaluate their relationship. Mutual dependence was quantified to assess the strength of the relationship between each feature and the target variable. Initially, the features and target variable were discretized to simplify the calculation of their probability distributions. Subsequently, the joint probability distribution P (X, Y) between the features and target variable, along with their marginal probability distributions P(X) and P(Y), were calculated. We computed the mutual information between each feature and the target variable by iterating over all possible values of the feature (x) and the target variable (y). Subsequently, the mutual-information formula was employed:


$$MI(X,Y)=\sum_{x\in X}\sum_{y\in Y}P(x,y)log\frac{P(x,y)}{P(x)P(y)}$$

Features were prioritized based on their mutual information values, and the top 15 features by mutual information score were retained.

### 2.5 Machine-learning algorithms

**Figure 2:**
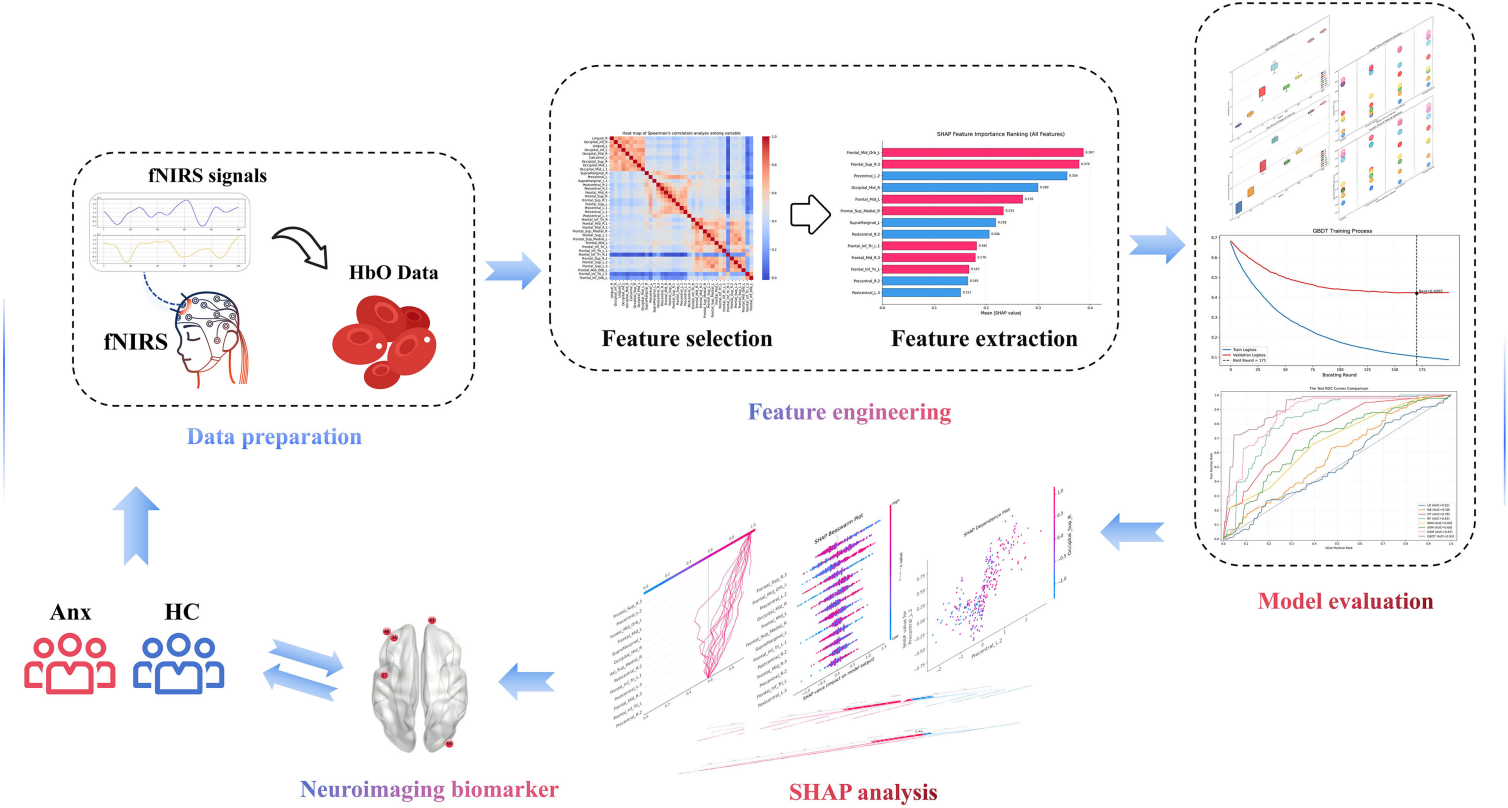
A machine-learning framework was developed to classify anxiety using fNIRS data. HbO signals were collected from the anxiety group and HC using fNIRS. After feature extraction and selection, the model was trained on resting-state data with hyperparameters optimized through cross-validation. SHAP analysis was applied for model interpretation and biomarker identification.

Machine learning was the key analytical approach used to develop a diagnostic tool with a focus on objectivity and interpretability. The tool’s purpose was to detect neuroimaging biomarkers in individuals with anxiety and shed light on patterns of brain activity and hemodynamic alterations associated with anxiety during rest-state. **Figure 2** illustrates the detailed framework and workflow.

This study utilized the Scikit-learn 1.6.1 machine-learning library in Python to optimize eight supervised classification algorithms: logistic regression (LR), naive Bayes (NB), support vector machine (SVM), decision tree (DT), K-nearest neighbors (KNN), random forest (RF), gradient boosting decision tree (GBDT), and gradient boosting machine (GBM). These algorithms were used to investigate associations between specific relevant features and their respective diagnostic results.

This study used a backward selection method in machine learning for feature selection. This regression-based technique was applied by initially fitting a model with all potential variables. We then iteratively eliminated the least significant variable based on partial F-tests, continuing this process until all remaining variables achieved statistical significance, thus obtaining the optimal model. Our selection criteria encompassed statistical tests, information criteria such as AIC, and performance metrics, which collectively reduced model complexity, enhanced robustness.

After feature engineering of the raw near-infrared spectroscopy (NIRS) data, a feature set X and a target variable y were obtained. Based on a training set and a test set that had been pre-divided into strata in a 7:3 ratio, various classification algorithms were applied with specified parameter ranges. Using GridSearch-CV with 5-fold cross-validation and negative log loss as the evaluation metric, the optimal parameter combination for the model was determined on the training set. To further improve generalization performance, an early stopping mechanism was introduced during training to determine the optimal number of iterations, effectively preventing overfitting.

### 2.6 Model evaluation

The model developed yielded the following indicators: True positive (TP) and true negative (TN) denoted correct predictions of anxious patients as anxious and HC as non-anxious, respectively. False positives (FP) and false negatives (FN) represented misclassification of HC as anxious and participants with anxiety as HC, respectively.

Accuracy: gauged the overall correctness of model predictions, computed as (TP+TN)/(TP+TN+FP+FN). Sensitivity (Recall): quantified the proportion of correctly predicted participants with anxiety among all actual participants with anxiety, expressed as TP/(TP+FN). Specificity: measured the proportion of correctly predicted HC among all actual HCs, calculated as TN/(FP+TN). Precision: assessed the proportion of correctly predicted participants with anxiety among all predicted participants with anxiety, determined by TP/(TP+FP). The F1 score, harmonic mean of precision and recall, was given by 2TP/(2TP+FP+FN). ROC curve illustrated the model’s classification performance across various thresholds, with the false positive rate (1-specificity) and true-positive rate (sensitivity) on the x-axis and y-axis, respectively. The area under the curve (AUC) values, which ranged from 0.5–1.0, represented the area under the ROC (AUC-ROC) curve, and higher values indicated greater diagnostic accuracy.

Based on these metrics, a comprehensive evaluation strategy was implemented to assess both model stability and interpretability. Model performance was evaluated using five−fold stratified cross−validation. In each fold, the model was trained on four folds and evaluated on the remaining fold. The final performance metrics are reported as mean ± standard deviation across the five folds. To further assess the stability of the model on the independent test set, bootstrap resampling (1000 replicates) was performed, and the mean ± standard deviation of each metric are presented.

In addition to quantitative performance, the interpretability of the model was examined to gain deeper insights into its decision−making process. The SHAP method assessed the significance of the features, and a higher absolute SHAP value indicated a more pronounced effect on the prediction score. This method offered both global and local interpretations to explain the model. Global interpretation provided accurate and uniform attribution values for each feature and elucidated the relationship between the input features and anxiety. Conversely, local interpretation analyzed specific data inputs to provide detailed predictive outcomes for individual instances.

To complement the analysis, various visual interpretations, such as box plots of model performance metrics, bubble plots, log loss variation curves, receiver operating characteristic (ROC) curves, SHAP importance ranking, swarm, decision plots, heatmap and partial dependence plots, were employed.

## 3 Results

### 3.1 Selection and comparison of multiple machine-learning models

The results of the demographic are presented in **Supplementary Table 1**. Spearman’s correlation coefficient analysis yielded P-values and feature-feature high-correlation pairs used for collinearity screening (**Supplementary Table 2**). After feature selection, the correlations among features calculated on the train set are shown in **Figure 1D**, and the top 20 channel features were ultimately selected to construct the initial feature set (**Supplementary Table 3**). Subsequently, backward selection was applied within each classification model to refine its own optimal feature subset for training and testing.

**Figure 3:**
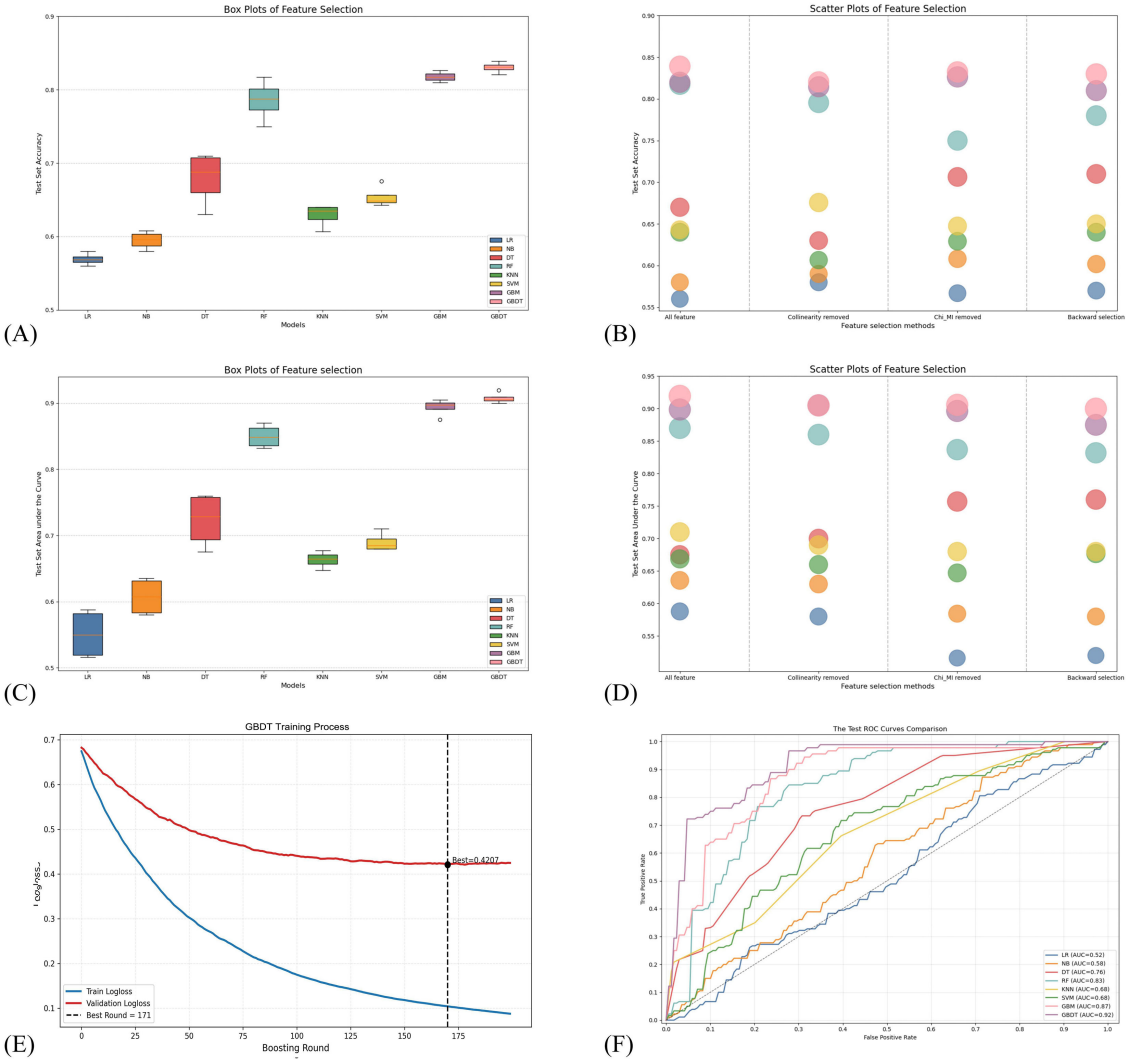
Eight machine-learning model performance metrics. **(A, C)** Box plots showing test-set accuracy and AUC across models. **(B, D)** Scatter plots showing the distribution of test-set accuracy and AUC across feature-selection strategies. **(E)** Training and validation log-loss curves for the final model. **(F)** ROC curves of the eight classifiers on the independent test set.

**Figures 3A, C** present the test set accuracy and AUC values for each model across varying quantities of feature selections via box plots. **Figures 3B, D** present the fluctuations in the test set accuracy and AUC among the eight models across the different feature selection methods. The GBDT model consistently exhibited superior predictive performance throughout the feature selection process, with the optimal feature set incorporated at its peak performance evaluation stage: “Frontal_Mid_L”, “Frontal_Sup_Medial_R”, “Precentral_L.2”, “Postcentral_L.3”, “Postcentral_R.2”, “Frontal_Inf_Tri_L.1”, “Frontal_Mid_R.3”, “Precentral_R.2”, “Frontal_Inf_Tri_L”, “Occipital_Mid_R”, “Frontal_Mid_Orb_L”, “SupraMarginal_L”, “Frontal_Sup_R.3”. **Supplementary Table 4** presents the composition of the channel variable.

Our statistical analysis successfully identified effective NIRS time-domain features correlated with anxiety, which enhanced the precision of feature representation in model identification. We observed that as the uncertainty of the random variable X concerning the target variable y decreased, their mutual dependence strengthened. This indicated that these features were more likely to be associated with the predicted outcomes, which further validated the effectiveness of the mutual information approach for feature selection. Consequently, these features were most pertinent for predicting outcomes.

Different analytical methods led to variations in the performance metrics of the eight models. After comprehensive comparison, the GBDT model exhibited the best performance. As shown in **Figures 3B, D**, although mutual information analysis conducted after chi-square tests did not yield a statistically significant improvement in model performance, it substantially reduced the number of features. Ultimately, the models achieved comparable performance with fewer than 15 features to that obtained with the full feature set, indicating that a small set of informative features can effectively characterize anxiety-related brain activity. This underscores the critical role of feature selection in optimizing diagnostic tools and identifying core biomarkers.

### 3.2 Performance metrics for multiple machine-learning models

We systematically evaluated and compared multiple classifiers, analyzing performance metrics for anxiety prediction in the test dataset, as detailed in **Table 1**. Our findings highlighted the superior predictive capability of the GBDT classification model, which excelled in accuracy and maintained a favorable tradeoff between false-negative and false-positive rates.

**Table 1:** Performance metrics of the eight models with different amounts of features.

Models	LR	NB	DT	RF	KNN	SVM	GBM	GBDT
Feature	15	8	15	14	12	13	11	13
Accuracy	0.567 ± 0.025	0.602 ± 0.026	0.706 ± 0.025	0.777 ± 0.023	0.637 ± 0.026	0.645 ± 0.026	0.809 ± 0.022	0.826 ± 0.020
AUC	0.516 ± 0.031	0.578 ± 0.031	0.757 ± 0.027	0.832 ± 0.023	0.677 ± 0.029	0.677 ± 0.029	0.875 ± 0.020	0.900 ± 0.017
Precision	0.581 ± 0.029	0.595 ± 0.029	0.737 ± 0.032	0.737 ± 0.028	0.677 ± 0.035	0.637 ± 0.030	0.818 ± 0.028	0.798 ± 0.027
F1-score	0.668 ± 0.024	0.712 ± 0.023	0.734 ± 0.026	0.823 ± 0.020	0.669 ± 0.028	0.723 ± 0.024	0.830 ± 0.021	0.855 ± 0.018
Sensitivity	0.788 ± 0.029	0.889 ± 0.023	0.733 ± 0.033	0.932 ± 0.018	0.662 ± 0.035	0.839 ± 0.027	0.844 ± 0.027	0.921 ± 0.020
Specificity	0.291 ± 0.037	0.243 ± 0.036	0.673 ± 0.039	0.584 ± 0.041	0.605 ± 0.041	0.402 ± 0.042	0.765 ± 0.035	0.709 ± 0.038

Cross-validation results illustrated that the GBDT model, which incorporated 13 feature patterns, effectively differentiated between patients with anxiety and HC. Hyperparameters, determined through a grid search, were learning rate=0.05, maximum tree depth=7, subsample=0.7, number of trees=200, and optimal number of iterations=171 (**Figure 3E**).

**Figure 3F** presents the ROC curves of the eight machine-learning models on the test set. The GBDT model, employing 13 features, achieved the highest predictive performance with an AUC of 0.900, followed closely by the GBM model (11 features, AUC = 0.875). In anxiety diagnosis, minimizing false negatives is critical, as missed detections may delay intervention and exacerbate clinical outcomes. The ROC curve demonstrates that GBDT maintained a high true-positive rate across various thresholds while keeping the false-positive rate within an acceptable range—a key consideration for diagnostic tools.

Regarding stability, all models exhibited small standard deviations (mostly 0.02–0.04) across five-fold cross-validation, indicating robust performance. Notably, GBDT showed the smallest deviations overall (AUC SD = 0.017), further confirming its reliability. Although random forest exhibited exceptionally high sensitivity, its relatively low specificity may lead to an increased false-positive rate.

### 3.3 Interpretation of machine-learning models

**Figure 4:**
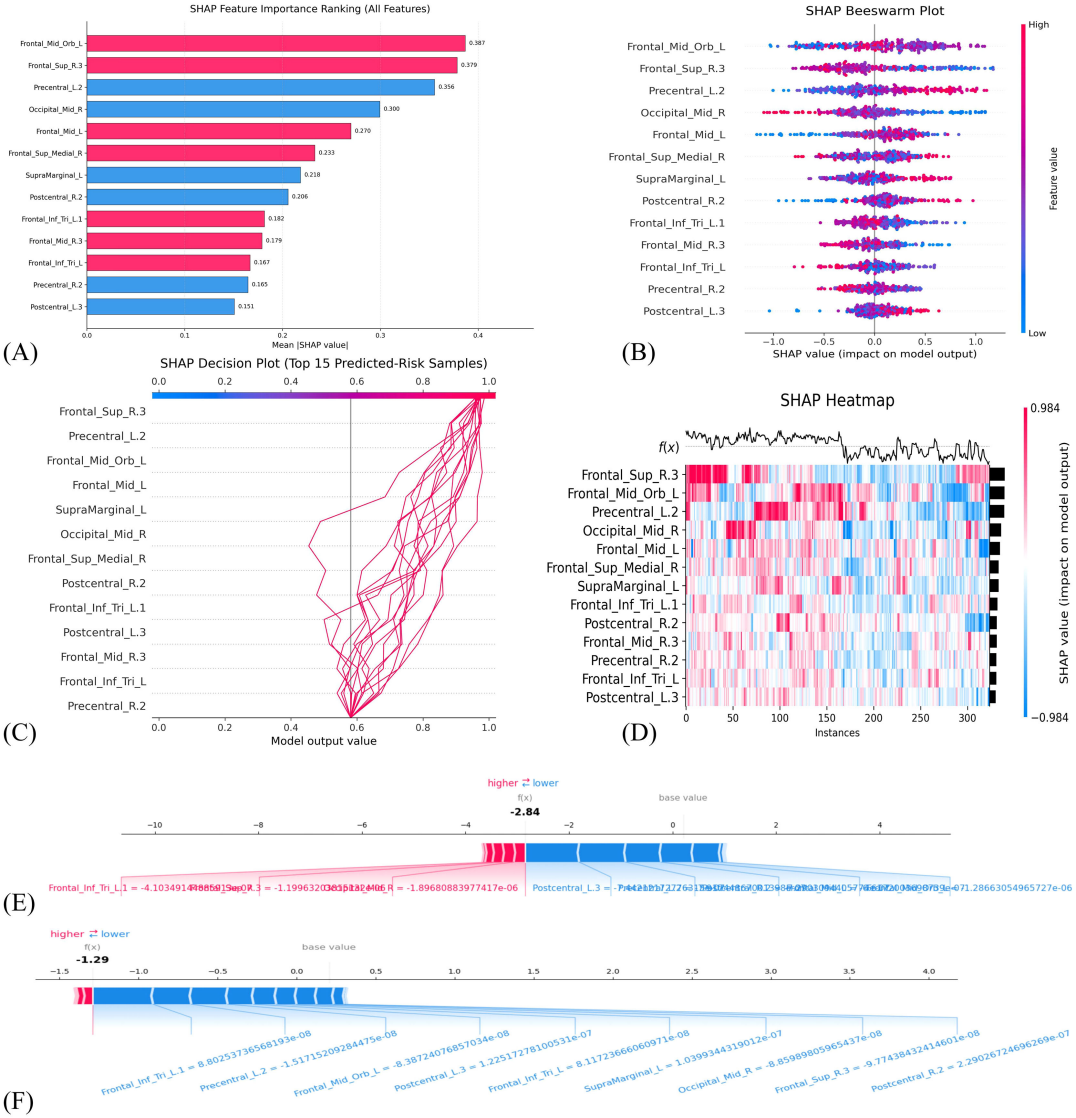
SHAP-based interpretation of the final model. **(A)** Mean absolute SHAP importance ranking. **(B)** SHAP beeswarm plot. **(C)** SHAP decision plot. **(D)** SHAP heatmap. **(E, F)** Force plots showing individual risk predictions for two representative samples.

**Figure 5:**
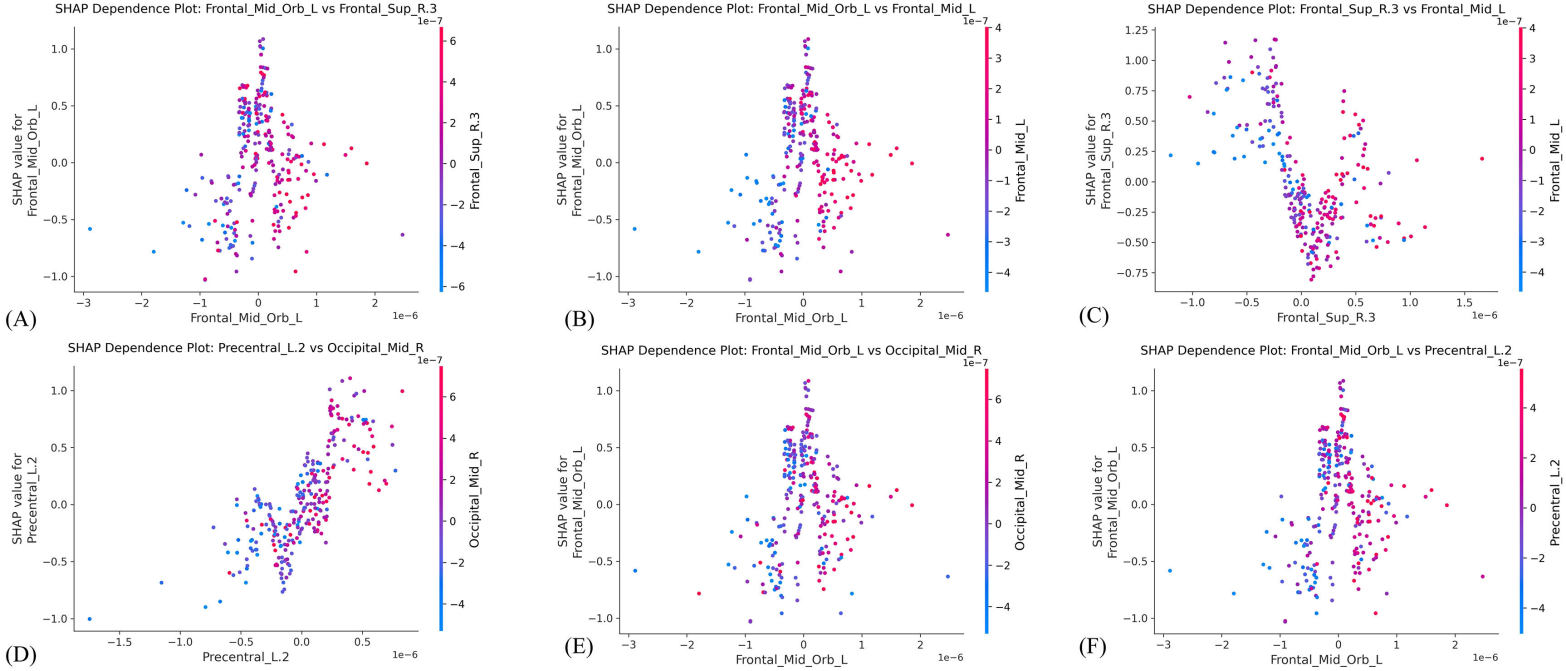
Pairwise SHAP dependence plots among selected top-ranked features. **(A, B)** Dependence of the orbitofrontal cortex channels on two other prefrontal cortex channels **(C)** Dependence between the dorsolateral prefrontal cortex and middle frontal cortex channels. **(D)** Dependency between the motor cortex and visual cortex channels **(E, F)** Dependence of the orbitofrontal cortex channels on motor cortex and visual cortex channels.

To enhance the interpretability of our model, we performed a detailed analysis of the GBDT algorithm results via the SHAP method. We computed the SHAP values rooted in game theory and quantified the influence of each feature on the model’s output. A positive and negative SHAP value for predicting anxiety signified a feature’s pivotal role in determining the anxiety category and the opposite scenario, respectively. **Figure 4A** illustrates the assessment of feature importance using mean absolute SHAP values and visually represents the relative significance of each feature in anxiety prediction. Distribution of high and low feature concentrations, along with the SHAP values of features across the dataset, elucidated the varying contributions of features to anxiety prediction, as presented in **Figures 4B, C** illustrates the incremental impact of each feature on its predictive capacity from the baseline, which collectively shaped the final prediction. **Figure 4D** visualizes the direction and magnitude of each feature’s contribution to the model’s prediction across individual samples. **Figures 4E, F** exhibit how changes in feature values markedly influence the ultimate risk score and display personalized risk prediction trajectories for two patients with typical anxiety. **Figure 5** primarily elucidates the non-linear associations between the features and the target variable and reciprocal regulatory relationships among different features.

## 4 Discussion

This study proposes and validates a biomarker-guided classification framework based on machine learning and resting-state functional near-infrared spectroscopy (fNIRS). Employing a case-control design with 50 anxiety disorder patients and 40 healthy controls in emerging adulthood, the framework extracts time-domain HbO features significantly correlated with anxiety, forming a unique pattern enabling neural signal differentiation without task stimuli. We rigorously evaluated the framework’s efficacy, and the GBDT model showed the best performance among the evaluated models. SHAP analysis further revealed key predictive contributions from specific prefrontal cortical regions, highlighting their importance as core neurophysiological markers for anxiety classification.

Our SHAP feature importance analysis revealed that the machine-learning model’s discriminative capacity between patients with anxiety and HC was primarily driven by the top five neurophysiological features that exceeded the mean absolute SHAP value threshold (> 0.25). The highest-ranked feature channel, “Frontal_Mid_Orb_L”, anatomically aligned with the left orbitofrontal cortex (OFC), which demonstrated strong association with affective regulation processes, value-based decision-making under uncertainty, and social cognition. This region exhibits heightened activation during the comparative reward-punishment appraisal of behavioral choices and processing of socially salient stimuli. The second-most influential channel, “Frontal_Sup_R.3”, corresponded to the right dlPFC, which reflected this region’s established role in cognitive control mechanisms, sustained attentional processing, and working memory maintenance. The third-ranked feature channel, “Precentral_L.2”, was situated adjacent to the left precentral gyrus (PreCG), which was associated with the motor cortex, responsible for coordinating movements of the hands and face, and regulating respiration and spontaneous blinking. The fourth-ranked feature channel, “Occipital_Mid_R”, was positioned near the right medius occipital gyrus (MOG), part of the visual cortex region primarily engaged in processing visual, auditory, and somatic stimuli. The fifth-ranked feature channel, “Frontal_Mid_L”, corresponds anatomically to the left middle frontal gyrus (MFG), a region that is a major component of the dlPFC and plays a central role in executive control and top-down emotional regulation.

This study found that anxiety states were associated with reduced hemodynamic signals in the OFC, while features related to the dlPFC showed an increased predictive contribution. This pattern is consistent with previous neuroimaging studies, which indicate that anxiety disorders are characterized by impaired functioning of the emotional regulation network (mediated by the OFC), accompanied by compensatory overactivation of the cognitive control circuit (mediated by the dlPFC) (26). We infer that the enhanced involvement of the dlPFC may represent a compensatory response to OFC dysfunction; however, this compensatory activation appears insufficient to restore emotional homeostasis and may instead reflect a maladaptive prefrontal imbalance.

Furthermore, the analysis revealed altered functional coupling between prefrontal regions and the sensorimotor cortex. Specifically, the left PreCG exhibited significant reciprocal dependence with occipital visual regions, while the PreCG itself demonstrated abnormal functional connectivity with emotion-regulating regions. These findings are consistent with previous literature (27), which indicates that anxiety is associated with increased motor cortex excitability at rest, and that elevated functional connectivity in the PreCG has been observed in patients with generalized anxiety disorder and panic disorder. The reduced involvement of the right MOG is consistent with observations that anxiety is associated with alterations in visual processing and weakened sensory responses to external stimuli.

Our findings aligned closely with those of a prior investigation on the interplay between prefrontal cortex (PFC) functionality and emotion regulation (28). This study highlighted the PFC’s role in modulating anxiety in primates and attributed this capability to its evolutionary expansion, which equipped primates with sophisticated regulatory mechanisms to manage anxiety. Notably, among the top five features identified by the SHAP mean values in our study, pivotal PFC regions represented a significant portion (≥ 60%), and specifically encompassed the left orbito, right dorsolateral, and left medial frontal areas. These three functional regions, integral to the PFC core, play crucial roles in anxiety-related processing, as supported by their functional connectivity and laminar architecture. Hence, resting-state investigations can reveal inherent functional connectivity and spontaneous brain activity patterns without external task influences, a conclusion corroborated by various independent studies and considered highly valuable.

Previous neuroimaging studies of anxiety disorders have predominantly employed fMRI to investigate task-related brain activation patterns, whereas studies using fNIRS remain comparatively limited. However, with advances in research on mental disorders, recent fNIRS-based investigations of anxiety and other psychiatric conditions, along with the exploration of potential biomarkers, have garnered increasing interest. A study developed a convolutional neural network (CNN) architecture for major depressive disorder (MDD) classification via a limited fNIRS dataset (48 patients with MDD vs. 68 HC) acquired during a Stroop task paradigm (29). The optimal model demonstrated a classification performance with 84.48% accuracy, 83.37% sensitivity, and 85.29% specificity. CNNs exhibited superior capacity in balancing sensitivity-specificity trade-offs. In a complementary investigation, Mao et al. systematically explored neuroimaging biomarkers for mental disorder identification via an expanded cohort (289 MDD patients vs. 178 HC) under a verbal fluency task (VFT) paradigm (30). Through multi-model machine-learning analysis, their GBDT classifier, which utilized 180 optimized features, achieved peak performance metrics of 82.9 ± 5.3% accuracy, 91.4 ± 0.51% sensitivity, and 68.2 ± 16.2% specificity, which demonstrated the feasibility of feature-driven approaches for psychiatric biomarker discovery.

Previous studies have demonstrated the detection of mental disorders via task-based fNIRS, the standardization of task validity across diverse participants remains challenging due to individual differences in comprehension, motivation, and attention. In contrast, our study focused on the resting state, which provides a more stable measure of intrinsic brain activity where inter-individual differences primarily reflect fundamental physiological characteristics. Despite the potential of this approach, research utilizing resting-state fNIRS for anxiety remains underdeveloped compared to the well-established role of resting-state fMRI in mapping neural networks and functional abnormalities (31). Consequently, the methodological advantages and clinical potential of resting-state fNIRS in psychiatry await dedicated exploration.

Recent fNIRS-based resting-state functional connectivity (rsFC) research has advanced our comprehension of anxiety disorders. A neuroimaging study built a network with 36 nodes and 630 edges, and compared rsFC between patients with anxiety and HC (32), and uncovered unique prefrontal topological traits and predictive value of prefrontal adjacent channels for anxiety detection. Additionally, a team at Wuhan University People’s Hospital created a machine-learning framework via region-specific resting-state neuroimaging features (33), which reached an AUC of 0.802. It also distinguished frontal-temporal rsFC patterns in patients with anxiety and depression versus HCs. These studies confirmed that fNIRS-constructed rsFC networks were feasible and useful for machine-learning identification of anxiety-depression spectrum disorders. Malfunction of the prefrontal cortex contributes to psychiatric disorders, which aligns with established neuroscience theories and further underscores the critical involvement of brain regions, such as the frontal and temporal lobes, in conditions related to emotion and cognition.

Although this study offers theoretical insights into psychopathology through dichotomous resting-state fNIRS analysis, its clinical utility remains constrained by high psychiatric comorbidity rates. Overlapping psycho-behavioral manifestations and neurobiological signatures across disorders obscure anxiety-specific pathophysiological mechanisms (34). Shen et al. (2025) employed a task-based fNIRS with deep learning to probe prefrontal cortex (PFC) dysfunction in MDD, GAD, and comorbidities (35). Their model achieved a 77.19% ternary classification accuracy, and the right orbitofrontal cortex demonstrated discriminative power (AUC = 0.83). Notably, the left ventromedial PFC activation patterns effectively distinguished pure GAD from comorbid GAD-MDD, which suggested the use of neurofunctional biomarkers for differential diagnosis.

This study has several limitations. First, the model was developed and validated on a modest-sized, single-site cohort of young adults with elevated anxiety symptoms, not a clinically diagnosed population. This limits the generalizability of our findings, and external validation in independent, multi-center clinical cohorts is needed to confirm the robustness of this preliminary classifier. Second, although we excluded participants with major comorbidities and medication use, other factors such as subclinical symptoms, lifestyle habits, and family psychiatric history were not fully measured (36). Their potential influence on the identified neurophysiological features requires further investigation.

Future research should build on these findings by addressing these limitations. Including participants from multiple centers and incorporating those with comorbid conditions will be crucial for validating the model’s generalization performance and for better understanding disorder-specific neural signatures. Furthermore, integrating multimodal data—such as combining task-state fNIRS, detailed clinical histories, and longitudinal designs—may improve diagnostic accuracy and help evaluate the prognostic value of the identified biomarkers.

## 5 Conclusion

In conclusion, this study demonstrates the feasibility of distinguishing individuals with elevated anxiety from healthy controls using resting-state fNIRS signals. The identified multiple HbO-based features serve as candidate biomarkers that merit further investigation. Although currently in its preliminary stages and requiring validation in larger clinical cohorts, this work demonstrates the potential of integrating fNIRS with explainable artificial intelligence, promising to advance the development of more objective assessment tools in psychiatry.

## Data Availability

The original contributions presented in the study are included in the article/**Supplementary Material**. Further inquiries can be directed to the corresponding author.
