# Supplementary material for: Identifying neuroimaging biomarkers for anxiety in emerging adults using machine learning and functional near-infrared spectroscopy during resting-state
Source: Front Psychiatry. 2026 Apr 10;17:1722529. doi: 10.3389/fpsyt.2026.1722529 (PMC13106591; doi:10.3389/fpsyt.2026.1722529)
Supplement: Supplementary file 1 [file Table1.docx]

Supplementary Material

# Supplementary Tables

## Supplementary Table S1

Results of difference tests for demographics and psychological assessments.

| Characteristic | Anx(n=50) | HC(n=40) | Statistic | p-value | Effect Size |
| --- | --- | --- | --- | --- | --- |
| Age | 18.64 ± 0.78 | 18.67 ± 0.62 | Z = 0.630 | 0.529 | r = 0.066 |
| Sex, Male | 20 (40.0%) | 13 (32.5%) | χ² = 0.437 | 0.508 | C = 0.665 |
| Education, years | 12.12 ± 0.48 | 12.08 ± 0.67 | Z = 0.114 | 0.909 | r = 0.012 |
| GAD-7 | 8.98 ± 3.22 | 2.08 ± 1.14 | Z = 8.172 | <0.01 | r = 0.861 |
| Smoking/Alcohol (Positive) | 0 (0.0%) | 0 (0.0%) | / | / | / |
| Family history (Positive) | 0 (0.0%) | 0 (0.0%) | / | / | / |

## Supplementary Table S2

Spearman correlation coefficients and P-value table

|  | **Feature** | **Correlation** | **P-Value** |
| --- | --- | --- | --- |
| 0 | Lingual_R | 0.000 | 0.851 |
| 1 | Occipital_Inf_R | -0.004 | 0.018 |
| 2 | Lingual_L | 0.000 | 0.929 |
| 3 | Occipital_Inf_L | -0.003 | 0.120 |
| 4 | Occipital_Mid_R | 0.007 | 0.000 |
| 5 | Calcarine_L | 0.011 | 0.000 |
| 6 | Occipital_Sup_R | 0.006 | 0.000 |
| 7 | Occipital_Mid_L | 0.005 | 0.003 |
| 8 | Occipital_Mid_L.1 | -0.001 | 0.459 |
| 9 | SupraMarginal_R | -0.002 | 0.250 |
| 10 | Postcentral_R | -0.004 | 0.023 |
| 11 | Postcentral_R.1 | -0.005 | 0.003 |
| 12 | Precentral_R | -0.010 | 0.000 |
| 13 | Precentral_R.1 | -0.004 | 0.017 |
| 14 | Precentral_L | 0.000 | 0.769 |
| 15 | Postcentral_L | -0.007 | 0.000 |
| 16 | Postcentral_L.1 | 0.000 | 0.782 |
| 17 | SupraMarginal_L | -0.001 | 0.614 |
| 18 | SupraMarginal_L.1 | 0.007 | 0.000 |
| 19 | Postcentral_R.2 | 0.009 | 0.000 |

## Continued Supplementary Table S2

Spearman correlation coefficients and P-value table

|  | **Feature** | **Correlation** | **P-Value** |
| --- | --- | --- | --- |
| 20 | Precentral_R.2 | -0.001 | 0.696 |
| 21 | Frontal_Mid_R | -0.001 | 0.726 |
| 23 | Frontal_Sup_R.1 | -0.001 | 0.583 |
| 24 | Frontal_Sup_L | -0.003 | 0.073 |
| 25 | Precentral_L.1 | 0.011 | 0.000 |
| 26 | Precentral_L.2 | 0.001 | 0.883 |
| 27 | Postcentral_L.2 | 0.001 | 0.409 |
| 28 | Postcentral_L.3 | 0.012 | 0.000 |
| 29 | Frontal_Inf_Tri_R | 0.001 | 0.729 |
| 30 | Frontal_Mid_R.1 | 0.002 | 0.381 |
| 31 | Frontal_Mid_R.2 | -0.006 | 0.001 |
| 32 | Frontal_Sup_Medial_R | 0.002 | 0.065 |
| 33 | Frontal_Sup_L.1 | 0.003 | 0.129 |
| 34 | Frontal_Sup_Medial_L | 0.003 | 0.223 |
| 35 | Frontal_Mid_L | -0.001 | 0.468 |
| 36 | Frontal_Inf_Tri_L | -0.005 | 0.006 |
| 37 | Frontal_Inf_Tri_L.1 | 0.005 | 0.004 |
| 38 | Frontal_Inf_Tri_R.1 | 0.010 | 0.000 |
| 39 | Frontal_Inf_Orb_R | 0.008 | 0.000 |
| 40 | Frontal_Sup_R.2 | 0.001 | 0.697 |

## Continued Supplementary Table S2

Spearman correlation coefficients and P-value table

|  | **Feature** | **Correlation** | **P-Value** |
| --- | --- | --- | --- |
| 41 | Frontal_Mid_R.3 | -0.004 | 0.043 |
| 42 | Frontal_Sup_R.3 | 0.002 | 0.377 |
| 43 | Frontal_Sup_L.2 | 0.003 | 0.083 |
| 44 | Frontal_Sup_L.3 | -0.002 | 0.245 |
| 45 | Frontal_Mid_Orb_L | 0.004 | 0.046 |
| 46 | Frontal_Inf_Tri_L.2 | 0.002 | 0.165 |
| 47 | Frontal_Inf_Orb_L | 0.006 | 0.002 |

|  |
| --- |

|  |
| --- |

|  |
| --- |

High-correlation features for screening

| **Feature_1** | **Feature_2** | **Abs_Spearman_r** | **Decision** |
| --- | --- | --- | --- |
| Precentral_R | Precentral_R.1 | 0.896312224 | drop Precentral_R (p2>p1) |
| Postcentral_L | Postcentral_L.1 | 0.847939089 | drop Postcentral_L (p2>p1) |
| Postcentral_R.1 | Precentral_R | 0.839230333 | drop Precentral_R (p1>p2) |
| Precentral_L | Postcentral_L | 0.836819539 | drop Postcentral_L (p1>p2) |
| Postcentral_R | Postcentral_R.1 | 0.835089178 | drop Postcentral_R.1 (p1>p2) |
| Frontal_Sup_Medial_L | Frontal_Sup_L.3 | 0.830990418 | drop Frontal_Sup_Medial_L (p2>p1) |
| Frontal_Mid_R | Frontal_Sup_R.1 | 0.829458231 | drop Frontal_Sup_R.1 (p1>p2) |
| Precentral_R.1 | Frontal_Mid_R | 0.8255624 | drop Precentral_R.1 (p2>p1) |
| Frontal_Sup_R.3 | Frontal_Sup_L.3 | 0.803959143 | drop Frontal_Sup_L.3 (p1>p2) |
| Precentral_R.2 | Frontal_Sup_R.1 | 0.803226783 | drop Frontal_Sup_R.1 (Deleted earlier) |

## Supplementary Table S3

Feature evaluation metrics under different statistical methods

| **Feature** | **VIF** | **Chi2_Score** | **Chi2_PValue** | **MI_Score** |
| --- | --- | --- | --- | --- |
| Frontal_Mid_L | 2.812 | 0.004 | 0.947 | 0.195 |
| Frontal_Sup_R.3 | 4.902 | 0.001 | 0.973 | 0.185 |
| Occipital_Mid_R | 3.668 | 0.084 | 0.772 | 0.178 |
| Frontal_Sup_L.2 | 3.872 | 0.001 | 0.979 | 0.169 |
| Postcentral_R.2 | 4.773 | 0.052 | 0.820 | 0.167 |
| Occipital_Mid_L.1 | 3.094 | 0.000 | 0.999 | 0.165 |
| Occipital_Sup_R | 2.958 | 0.084 | 0.772 | 0.160 |
| Frontal_Inf_Tri_L.1 | 3.136 | 0.010 | 0.922 | 0.159 |
| Frontal_Mid_R.3 | 4.480 | 0.001 | 0.973 | 0.158 |
| Precentral_L.2 | 2.516 | 0.026 | 0.872 | 0.156 |
| Frontal_Inf_Tri_L | 3.554 | 0.015 | 0.902 | 0.150 |
| Occipital_Inf_L | 3.764 | 0.218 | 0.641 | 0.150 |
| Frontal_Mid_Orb_L | 3.358 | 0.023 | 0.879 | 0.148 |
| SupraMarginal_L | 2.171 | 0.025 | 0.874 | 0.147 |
| Postcentral_L.3 | 3.056 | 0.066 | 0.798 | 0.145 |
| Occipital_Mid_L | 3.530 | 0.222 | 0.637 | 0.144 |
| Postcentral_R | 3.804 | 0.072 | 0.788 | 0.144 |
| Frontal_Sup_Medial_R | 4.902 | 0.041 | 0.840 | 0.143 |
| Precentral_R.2 | 4.638 | 0.003 | 0.959 | 0.143 |
| Lingual_R | 3.424 | 0.028 | 0.866 | 0.137 |
| Precentral_L | 3.095 | 0.104 | 0.747 | 0.135 |
| Frontal_Mid_R.1 | 4.072 | 0.056 | 0.813 | 0.135 |
| Frontal_Inf_Tri_R | 3.338 | 0.008 | 0.930 | 0.135 |
| Frontal_Inf_Tri_R.1 | 2.444 | 0.262 | 0.609 | 0.130 |
| Frontal_Sup_L | 1.713 | 0.001 | 0.977 | 0.130 |
| Frontal_Inf_Tri_L.2 | 2.350 | 0.082 | 0.775 | 0.128 |
| Frontal_Mid_R | 4.804 | 0.009 | 0.926 | 0.128 |
| Frontal_Sup_L.1 | 3.583 | 0.016 | 0.900 | 0.127 |

## Continued Supplementary Table S3

Feature evaluation metrics under different statistical methods

| **Feature** | **VIF** | **Chi2_Score** | **Chi2_PValue** | **MI_Score** |
| --- | --- | --- | --- | --- |
| Calcarine_L | 3.090 | 0.021 | 0.886 | 0.105 |
| Frontal_Sup_L.3 | 3.684 | 0.053 | 0.818 | 0.099 |
| Precentral_L.1 | 3.965 | 0.027 | 0.870 | / |
| SupraMarginal_L.1 | 3.266 | 0.020 | 0.888 | / |
| SupraMarginal_R | 3.129 | 0.023 | 0.881 | / |
| Frontal_Inf_Orb_L | 6.164 | / | / | / |
| Lingual_L | 5.901 | / | / | / |
| Frontal_Sup_R.2 | 5.838 | / | / | / |
| Occipital_Inf_R | 5.623 | / | / | / |
| Postcentral_L.2 | 5.535 | / | / | / |
| Frontal_Mid_R.2 | 5.414 |  |  |  |
| Frontal_Inf_Orb_R | 5.346 | / | / | / |

## Supplementary Table S4

48 Channels Anatomical label, Percentage of Overlap

| **Channel** | **Channel Name** | **Percentage Content** |
| --- | --- | --- |
| CH1 | Lingual_R, | 0.724 |
| CH1 | Occipital_Inf_R, | 0.069 |
| CH1 | Cerebelum_Crus1_R, | 0.207 |
|  |  |  |
| CH2 | Calcarine_R, | 0.199 |
| CH2 | Lingual_R, | 0.268 |
| CH2 | Occipital_Sup_R, | 0.010 |
| CH2 | Occipital_Mid_R, | 0.117 |
| CH2 | Occipital_Inf_R, | 0.406 |
|  |  |  |
| CH3 | Calcarine_L, | 0.012 |
| CH3 | Lingual_L, | 0.757 |
| CH3 | Occipital_Mid_L, | 0.019 |
| CH3 | Occipital_Inf_L, | 0.054 |
| CH3 | Cerebelum_Crus1_L, | 0.116 |
| CH3 | Cerebelum_Crus2_L, | 0.042 |
|  |  |  |
| CH4 | Calcarine_L, | 0.083 |
| CH4 | Lingual_L, | 0.230 |
| CH4 | Occipital_Mid_L, | 0.342 |
| CH4 | Occipital_Inf_L, | 0.345 |
|  |  |  |
| CH5 | Occipital_Sup_R, | 0.012 |
| CH5 | Occipital_Mid_R, | 0.976 |
| CH5 | Occipital_Inf_R, | 0.012 |
| CH6 | Calcarine_L, | 0.722 |
| CH6 | Lingual_L, | 0.025 |
| CH6 | Occipital_Mid_L, | 0.254 |
|  |  |  |
| CH7 | Calcarine_L, | 0.033 |
| CH7 | Calcarine_R, | 0.278 |
| CH7 | Cuneus_R, | 0.248 |
| CH7 | Occipital_Sup_R, | 0.440 |
|  |  |  |
| CH8 | Calcarine_L, | 0.123 |
| CH8 | Occipital_Sup_L, | 0.155 |
| CH8 | Occipital_Mid_L, | 0.723 |
|  |  |  |
| CH9 | Occipital_Mid_L, | 1.000 |
|  |  |  |
| CH10 | SupraMarginal_R, | 1.000 |
|  |  |  |
| CH11 | Postcentral_R, | 0.375 |
| CH11 | Parietal_Sup_R, | 0.030 |
| CH11 | Parietal_Inf_R, | 0.242 |
| CH11 | SupraMarginal_R | 0.352 |
|  |  |  |
| CH12 | Precentral_R, | 0.350 |
| CH12 | Postcentral_R, | 0.646 |
| CH12 | Parietal_Sup_R, | 0.004 |
|  |  |  |
| CH13 | Precentral_R, | 0.757 |
| CH13 | Postcentral_R, | 0.243 |
| CH14 | Precentral_R, | 0.858 |
| CH14 | Frontal_Sup_R, | 0.003 |
| CH14 | Postcentral_R, | 0.139 |
|  |  |  |
| CH15 | Precentral_L, | 0.615 |
| CH15 | Postcentral_L, | 0.359 |
| CH15 | Paracentral_Lobule_L, | 0.026 |
|  |  |  |
| CH16 | Precentral_L, | 0.478 |
| CH16 | Postcentral_L, | 0.522 |
|  |  |  |
| CH17 | Postcentral_L, | 0.860 |
| CH17 | Parietal_Inf_L, | 0.140 |
|  |  |  |
| CH18 | Postcentral_L, | 0.035 |
| CH18 | Parietal_Inf_L, | 0.364 |
| CH18 | SupraMarginal_L, | 0.601 |
|  |  |  |
| CH19 | Postcentral_L, | 0.006 |
| CH19 | SupraMarginal_L, | 0.978 |
| CH19 | Temporal_Sup_L, | 0.016 |
|  |  |  |
| CH20 | Precentral_R, | 0.190 |
| CH20 | Postcentral_R, | 0.810 |
|  |  |  |
| CH21 | Precentral_R, | 0.534 |
| CH21 | Frontal_Mid_R, | 0.331 |
| CH21 | Postcentral_R, | 0.135 |
|  |  |  |
| CH22 | Precentral_R, | 0.324 |
| CH22 | Frontal_Sup_R, | 0.031 |
| CH22 | Frontal_Mid_R, | 0.645 |
|  |  |  |
| CH23 | Precentral_R, | 0.098 |
| CH23 | Frontal_Sup_R, | 0.692 |
| CH23 | Frontal_Mid_R, | 0.210 |
| CH24 | Frontal_Sup_R, | 0.929 |
| CH24 | Supp_Motor_Area_R, | 0.071 |
|  |  |  |
| CH25 | Frontal_Sup_L, | 0.982 |
| CH25 | Frontal_Mid_L, | 0.018 |
|  |  |  |
| CH26 | Precentral_L, | 0.604 |
| CH26 | Frontal_Sup_L, | 0.209 |
| CH26 | Frontal_Mid_L, | 0.187 |
|  |  |  |
| CH27 | Precentral_L, | 0.788 |
| CH27 | Frontal_Mid_L, | 0.053 |
| CH27 | Postcentral_L, | 0.159 |
|  |  |  |

| CH28 | Precentral_L, | 0.245 |
| --- | --- | --- |
| CH28 | Postcentral_L, | 0.751 |
| CH28 | SupraMarginal_L, | 0.004 |
| CH29 | Precentral_L, | 0.029 |
| CH29 | Postcentral_L, | 0.971 |
|  |  |  |
| CH30 | Frontal_Mid_R, | 0.405 |
| CH30 | Frontal_Inf_Oper_R, | 0.015 |
| CH30 | Frontal_Inf_Tri_R, | 0.580 |
|  |  |  |
| CH31 | Frontal_Sup_R, | 0.005 |
| CH31 | Frontal_Mid_R, | 0.995 |
|  |  |  |
| CH32 | Frontal_Mid_R, | 0.851 |
| CH32 | Frontal_Inf_Tri_R, | 0.149 |
|  |  |  |
| CH33 | Frontal_Sup_R, | 0.450 |
| CH33 | Frontal_Mid_R, | 0.023 |
| CH33 | Frontal_Sup_Medial_L, | 0.008 |
| CH33 | Frontal_Sup_Medial_R, | 0.519 |
|  |  |  |
| CH34 | Frontal_Sup_L, | 0.709 |
| CH34 | Frontal_Mid_L, | 0.004 |
| CH34 | Frontal_Sup_Medial_L, | 0.287 |
|  |  |  |
| CH35 | Frontal_Sup_L, | 0.021 |
| CH35 | Frontal_Sup_Medial_L, | 0.662 |
| CH35 | Frontal_Sup_Medial_R, | 0.317 |
|  |  |  |
| CH36 | Frontal_Mid_L, | 1.000 |
| CH37 | Frontal_Mid_L, | 0.035 |
| CH37 | Frontal_Inf_Tri_L, | 0.965 |
|  |  |  |
| CH38 | Frontal_Mid_L, | 0.243 |
| CH38 | Frontal_Inf_Tri_L, | 0.757 |
|  |  |  |
| CH39 | Frontal_Inf_Oper_R, | 0.212 |
| CH39 | Frontal_Inf_Tri_R, | 0.788 |
|  |  |  |
| CH40 | Frontal_Mid_Orb_R, | 0.010 |
| CH40 | Frontal_Inf_Tri_R, | 0.382 |
| CH40 | Frontal_Inf_Orb_R, | 0.608 |
|  |  |  |
| CH41 | Frontal_Sup_R, | 0.771 |
| CH41 | Frontal_Mid_R, | 0.221 |
| CH41 | Frontal_Sup_Medial_R, | 0.007 |
|  |  |  |
| CH42 | Frontal_Sup_R, | 0.027 |
| CH42 | Frontal_Sup_Orb_R, | 0.023 |
| CH42 | Frontal_Mid_R, | 0.575 |
| CH42 | Frontal_Mid_Orb_R, | 0.375 |
|  |  |  |
| CH43 | Frontal_Sup_R, | 0.461 |
| CH43 | Frontal_Sup_Orb_R, | 0.201 |
| CH43 | Frontal_Sup_Medial_R, | 0.329 |
| CH43 | Frontal_Mid_Orb_R, | 0.009 |
|  |  |  |
| CH44 | Frontal_Sup_L, | 0.529 |
| CH44 | Frontal_Mid_L, | 0.471 |
|  |  |  |
| CH45 | Frontal_Sup_L, | 0.476 |
| CH45 | Frontal_Sup_Orb_L, | 0.189 |
| CH45 | Frontal_Sup_Medial_L, | 0.189 |
| CH45 | Frontal_Mid_Orb_L, | 0.147 |
|  |  |  |
| CH46 | Frontal_Sup_L, | 0.016 |
| CH46 | Frontal_Mid_L, | 0.304 |
| CH46 | Frontal_Mid_Orb_L, | 0.676 |
| CH46 | Frontal_Inf_Orb_L, | 0.004 |
|  |  |  |
| CH47 | Frontal_Inf_Oper_L, | 0.395 |
| CH47 | Frontal_Inf_Tri_L, | 0.602 |
| CH47 | Rolandic_Oper_L, | 0.003 |
|  |  |  |
| CH48 | Frontal_Inf_Tri_L, | 0.464 |
| CH48 | Frontal_Inf_Orb_L, | 0.536 |
